# Effects of FTMT Expression by Retinal Pigment Epithelial Cells on Features of Angiogenesis

**DOI:** 10.3390/ijms21103635

**Published:** 2020-05-21

**Authors:** Undral Buyandelger, Douglas G. Walker, Daijiro Yanagisawa, Toshifumi Morimura, Ikuo Tooyama

**Affiliations:** Molecular Neuroscience Research Center, Shiga University of Medical Science, Seta Tsukinowa-cho, Otsu, Shiga 520-2192, Japan; undral@belle.shiga-med.ac.jp (U.B.); walker@belle.shiga-med.ac.jp (D.G.W.); yanisagawa@belle.shiga-med.ac.jp (D.Y.); morimura@belle.shiga-med.ac.jp (T.M.)

**Keywords:** mitochondrial ferritin, vascular endothelial growth factor, angiogenesis, retinal pigment epithelium, age-related macular degeneration, differentiation

## Abstract

Aberrant angiogenesis is a pathological feature of a number of diseases and arises from the uncoordinated expression of angiogenic factors as response to different cellular stresses. Age-related macular degeneration (AMD), a leading cause of vision loss, can result from pathological angiogenesis. As a mutation in the mitochondrial ferritin (FTMT) gene has been associated with AMD, its possible role in modulating angiogenic factors and angiogenesis was investigated. FTMT is an iron-sequestering protein primarily expressed in metabolically active cells and tissues with high oxygen demand, including retina. In this study, we utilized the human retinal pigment epithelial cell line ARPE-19, both as undifferentiated and differentiated cells. The effects of proinflammatory cytokines, FTMT knockdown, and transient and stable overexpression of FTMT were investigated on expression of pro-angiogenic vascular endothelial growth factor (VEGF) and anti-angiogenic pigment epithelial-derived factor (PEDF). Proinflammatory cytokines induced FTMT and VEGF expression, while NF-κB inhibition significantly reduced FTMT expression. VEGF protein and mRNA expression were significantly increased in FTMT-silenced ARPE-19 cells. Using an in vitro angiogenesis assay with endothelial cells, we showed that conditioned media from FTMT-overexpressing cells had significant antiangiogenic effects. Collectively, our findings indicate that increased levels of FTMT inhibit angiogenesis, possibly by reducing levels of VEGF and increasing PEDF expression. The cellular models developed can be used to investigate if increased FTMT may be protective in angiogenic diseases, such as AMD.

## 1. Introduction

Aberrant or pathological angiogenesis is a feature of a number of degenerative diseases, including type-2 diabetes, stroke, and retinal diseases. One type of aberrant angiogenesis is choroidal neovascularization, where the proliferation of endothelial cells can lead to the formation of nonfunctional and leaking vessels [1]. Pathological angiogenesis is associated in these diseases with increased oxidative stress and inflammation, resulting in increased expression of pro-angiogenic growth factors, including vascular endothelial growth factor (VEGF), basic fibroblast growth factor (bFGF), and angiopoietins [2]. Age-related macular degeneration (AMD) is the major cause of vision impairment and blindness in elderly populations [3,4]. AMD is a multifactorial disease, in which both genetic and environmental factors have been implicated in its development [5,6]. Aging, inflammation, and hypoxia are considered the most important risk factors for AMD. The complex interactions of these factors lead to two distinct forms of AMD, geographic atrophic (dry) AMD and neovascular exudative (wet) AMD [7,8]. In atrophic dry AMD, both the retinal pigment epithelium (RPE) and photoreceptors gradually degenerate and the accumulation of cellular debris, known as drusen, occurs between the RPE cells and Bruch’s membrane [9,10]. In exudative AMD, relatively rapid visual loss can occur, with excessive expression of vascular endothelial growth factor (VEGF) by RPE cells. Increased VEGF expression in AMD can lead to proliferation of choroidal neovascularization and the presence of highly permeable vessels in the subretinal space [11,12]. In the retina, RPE cells secrete various growth factors and cytokines implicated in ocular diseases such as diabetic retinopathy, choroidal neovascularization, and macular edema [1]. In addition, tumor necrosis factor-α (TNF-α) expression has been found in high concentrations in the eyes of patients with AMD. Furthermore, TNF-α has been reported to induce the expression of VEGF in RPE cells, resulting in increased neovascularization in the exudative form of AMD [13,14,15].

Growing evidence suggests that free radical damage associated with age-related disorders, along with accumulation of iron, lipofuscin, and VEGF production are involved in the development of AMD [16,17,18,19]. Macular iron levels increase with age, and excess iron has been observed in RPE cells and Bruch’s membrane from postmortem subjects with AMD [20,21]. Recently, a *FTMT* gene mutation and resulting protein dysfunction were identified in a patient with AMD [22]. Mitochondrial ferritin (FTMT) is an iron-sequestering protein localized to the mitochondria and belongs to the ferritin family [23]. In general, FTMT expression is low in most cells and restricted to the testes, brain, heart, blood, and retina, tissues with high oxygen consumption [24,25,26]. In a previous study, we revealed that age-related increases in FTMT were involved in the regulation of cellular iron metabolism in murine RPE cells [27]. We also demonstrated that FTMT expression was increased in response to TNF-α via NF-κB activation in the human neuroblastoma cell line IMR-32 [28]. A number of studies have demonstrated that FTMT may have multiple properties, including protective roles against oxidative stress and hypoxia in neuronal cells [29,30,31,32,33]. Although expression of FTMT is usually very low to undetectable in most cell types, it is expressed at detectable levels in RPE cells [27]. The goal of this project was to examine the consequences of manipulating FTMT expression in RPE cells on expression of angiogenic factors including VEGF, and on angiogenesis using in vitro assays to model its potential role in AMD.

We compared differentiated and undifferentiated ARPE-19 cells to extend the relevance of this model for FTMT expression and investigated the consequences of inflammation, FTMT knockdown, and overexpression on separate features of angiogenesis. Key findings were reduction in VEGF expression and increased pigment epithelial-derived factor (PEDF) expression in RPE cells overexpressing FTMT. In addition, FTMT overexpression increased levels of mRNA for the RPE cell-differentiation marker retinal pigment epithelial-specific 65 kDa protein (RPE65). The effects of FTMT were evident in an in vitro angiogenesis assay, which demonstrated that conditioned media from FTMT-overexpressing cells significantly inhibited endothelial cell tube formation. Implications of these findings and future directions are discussed.

## 2. Results

### 2.1. FTMT Gene Expression in ARPE-19 Cells and Effects on Cell Differentiation

Optimal cellular models for human diseases employing cell lines use those that have retained many of the features of the primary cell type present in tissue. ARPE-19 cells are a spontaneously transformed proliferating cell line derived from human retina [34] that can be differentiated to a mature phenotype for experimental purposes but, in many previously published studies, have been used in the undifferentiated state [35,36,37]. As a foundation for this investigation, using the rapid differentiation protocol of Hazim et al. [37], we compared the expression of FTMT mRNA and characterized other phenotypic properties between undifferentiated and differentiated ARPE-19 cells.

**Figure 1:**
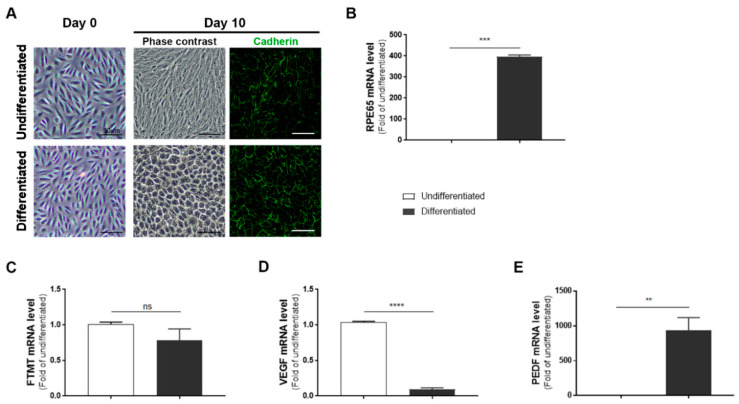
Features of undifferentiated and differentiated ARPE-19 cells: (**A**) Morphology of ARPE-19 cells maintained in growth media (undifferentiated) compared to cells maintained in nicotinamide-containing differentiation media (differentiated). The mature cobblestone morphology and immunoreactivity for the adhesion protein cadherin can be observed compared to the more disorganized morphology of undifferentiated cells. Scale bars represent 50 μm. (**B**) Relative expression of RPE65 mRNA in undifferentiated compared to differentiated cells: RPE65 mRNA expression was increased more than 350-fold. (*** *p* < 0.001, t test). (**C**) Mitochondrial ferritin (FTMT) mRNA was not significantly different between undifferentiated and differentiated cells (ns, not significant; t test). (**D**) Constitutive levels of vascular endothelial growth factor (VEGF) mRNA expression were significantly less in differentiated cells (***** *p* < 0.0001, t test). (**E**) Constitutive levels of pigment epithelial-derived factor (PEDF) mRNA expression were significantly increased in differentiated cells (** *p* < 0.01, t test). (**B**–**E**) Results of triplicate analyses of 2–3 independent experiments.

ARPE-19 cells after 10 days incubation in nicotinamide-containing differentiation media developed a “cobblestone” morphology with increased immunoreactivity for the junction protein cadherin (Figure 1A, day 10). The differentiated phenotype was confirmed by a 350-fold increase in expression of RPE65 mRNA, a specific marker for RPE cells, in differentiated compared to undifferentiated cells (Figure 1B). However, using the same samples, the expression of FTMT mRNA was decreased (though not significantly) in differentiated cells (Figure 1C) while the decreased expression of VEGF mRNA in differentiated cells (*p* < 0.0001, Figure 1D) and increased expression of PEDF mRNA (*p* < 0.01, Figure 1E) were significant.

### 2.2. Proinflammatory Cytokines Increased Levels of FTMT mRNA and VEGF mRNA and Protein in ARPE-19 Cells

**Figure 2:**
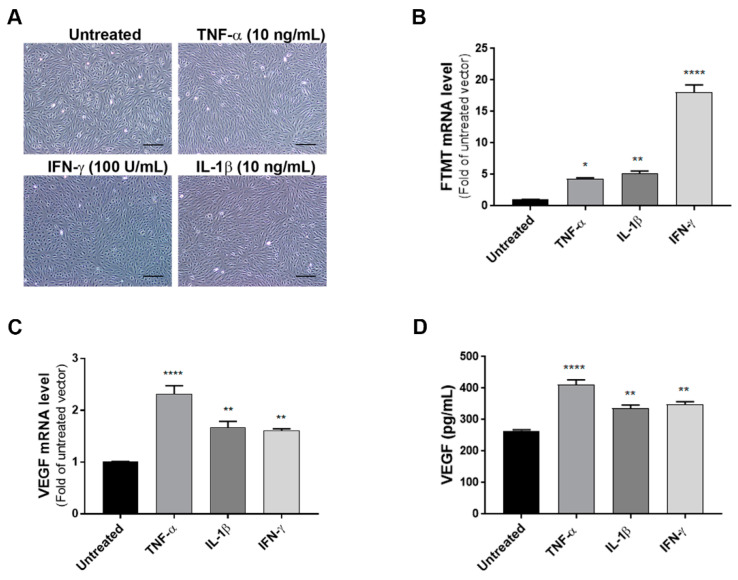
Effects of proinflammatory cytokines on the expression of FTMT and VEGF in ARPE-19 cells: (**A**) ARPE-19 cells were treated with TNF-α (10 ng/mL), IL-1β (10 ng/mL), and IFN-γ (100 U/mL) for 24 h. Scale bars represent 200 μm. (**B**,**C**) qPCR results showing the relative mRNA expressions of FTMT and VEGF mRNA expression results were normalized using ACTB. (**D**) Secreted VEGF in the conditioned media was measured using a VEGF ELISA: Data were analyzed by One-way ANOVA with Dunnett’s multiple comparison test. The results shown are the means ± SEM of four independent experiments (* *p* < 0.05; ** *p* < 0.01, and **** *p* < 0.0001).

As the levels of FTMT mRNA did not alter significantly in differentiated cells, some experiments were carried out using undifferentiated ARPE-19 cells as proliferating cells were more amenable to transient experimental manipulations. To define the effects of proinflammatory cytokines on the expression of VEGF and FTMT, ARPE-19 cells were treated with TNF-α (10 ng/mL), IL-1β (10 ng/mL) or IFN-γ (100 U/mL) for 24 h. Preliminary studies established these to be optimal doses (data not shown). Bright-field pictures depicting cellular morphology suggested that cytokine treatment at 24 h was not altering the morphological phenotype of these cells (Figure 2A). The lack of toxicity of proinflammatory cytokines on ARPE-19 cells was confirmed using a cell-viability/metabolic activity assay (Supplemental Figure S1). Using cytokine-treated cells, we next performed quantitative real-time reverse transcription polymerase chain reaction (qPCR) to measure mRNA expression of FTMT and VEGF. QPCR results demonstrated that mRNA levels of FTMT and VEGF were significantly increased in response to cytokine treatments compared with those in untreated cells (Figure 2B,C). Similar to our mRNA results, VEGF protein levels measured by ELISA in culture supernatants were significantly increased with cytokine treatments with greatest induction in cells treated with TNF-α (Figure 2D). This established that both FTMT and VEGF expression could be increased under proinflammatory conditions.

### 2.3. TNF-α Induced FTMT mRNA Expression Was Dependent on NF-κB Activation in ARPE-19 Cells

**Figure 3:**
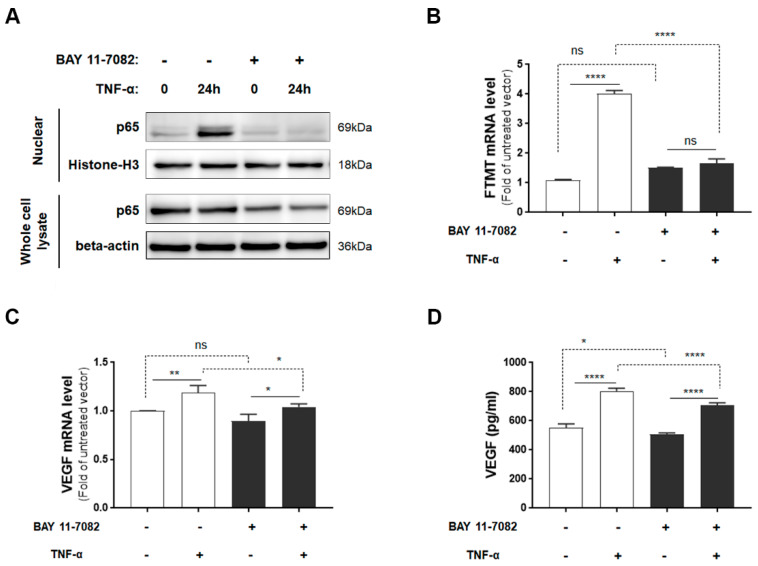
FTMT mRNA expression and VEGF protein secretion in response to TNF-α with or without NF-κB inhibitor BAY 11-7082: (**A**) Translocated levels of NF-κB subunit (p65) in nuclear extracts or in whole cell lysates after 24 h incubation with or without TNF-α (10 ng/mL) and BAY 11-7082 (5 μM). Relative levels of mRNA of (**B**) FTMT and (**C**) VEGF measured by qPCR and normalized by the ACTB gene in cells treated as in A (*n* = 4). (**D**) VEGF protein levels in conditioned media of cells after 24 h incubation with or without TNF-α (10 ng/mL) and BAY 11-7082 (5 μM) (*n* = 6). Data were analyzed by one-way ANOVA with the Sidak’s post hoc test (* *p* < 0.05; ** *p* < 0.01, and **** *p* < 0.0001). Results are shown as mean ± SEM.

Although IFN-γ increased FTMT mRNA expression to a greater level than TNF-α in ARPE-19 cells, as myeloid cells, not T-lymphocytes (source of IFN-γ), are observed in AMD and TNF-α has been implicated in AMD inflammation [15], our further studies employed TNF-α. As TNF-α is a potent activator of NF-κB [38] and TNF-α-mediated induction of FTMT in the neuronal IMR-32 cell line was modulated by NF-κB activation [28], we examined the effects of the NF-κB inhibitor BAY 11-7082 on both FTMT and VEGF expression. Initially, we determined the effects of BAY 11-7082 on undifferentiated ARPE-19 cell viability and showed that concentrations up to 5 µM did not exert significant toxicity on cells (Supplemental Figure S2). After 24 hours of treatment, TNF-α induced nuclear localization of p65, and this effect was effectively inhibited by BAY 11-7082 (5 µM) (Figure 3A). The induction of FTMT transcription by TNF-α was effectively inhibited by BAY 11-7082 in ARPE-19 cells, but this inhibition did not alter constitutive levels of FTMT mRNA (Figure 3B). Treatment of cells with BAY 11-7082 did not noticeably affect expression or secretion of constitutive levels of VEGF (Figure 3C,D), but there was a slight but statistically significant reduction in both VEGF mRNA and protein expression in NF-κB inhibited cells (Figure 3C,D).

### 2.4. VEGF Secretion Was Increased in FTMT Gene-silenced ARPE-19 Cells

**Figure 4:**
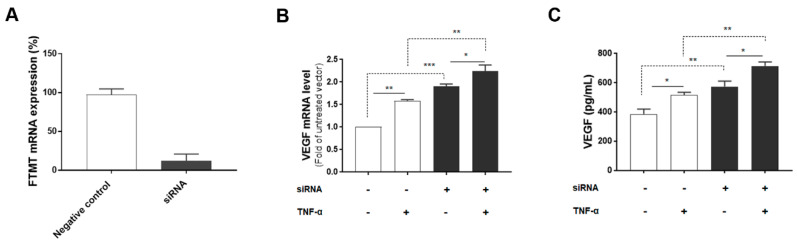
VEGF expression in FTMT gene-silenced ARPE-19 cells: (**A**) Effective silencing of the FTMT gene in ARPE-19 cells. Quantification of FTMT mRNA levels compared with the negative control group 48 h after FTMT siRNA transfection. Data were analyzed by t-test. (**B**) The qPCR results showing the amount of VEGF mRNA in FTMT silenced cells. The relative expression of mRNA was normalized by the ACTB gene. (**C**) Secreted VEGF in the conditioned media was measured using ELISA. Data for Figure 4B,C were analyzed by one-way ANOVA with the Sidak’s test (* *p* < 0.05 and ** *p* < 0.01, *** *p* < 0.001, *n* = 4). Results are shown as the mean ± SEM.

Since both FTMT and VEGF mRNA, and VEGF secretion were significantly upregulated by TNF-α in ARPE-19 cells, we next investigated the association between them by using siRNA to inhibit FTMT gene expression in ARPE-19 cells. Treatment of undifferentiated ARPE-19 cells with FTMT siRNA resulted in a greater than 90% decrease in FTMT mRNA compared to cells transfected with control siRNA (Figure 4A). Moreover, VEGF mRNA and protein levels were significantly increased in FTMT-silenced ARPE-19 cells compared to the control groups whether treated or not with TNF-α (Figure 4B,C).

### 2.5. FTMT Overexpression Reduced VEGF Production in ARPE-19 Cells

**Figure 5:**
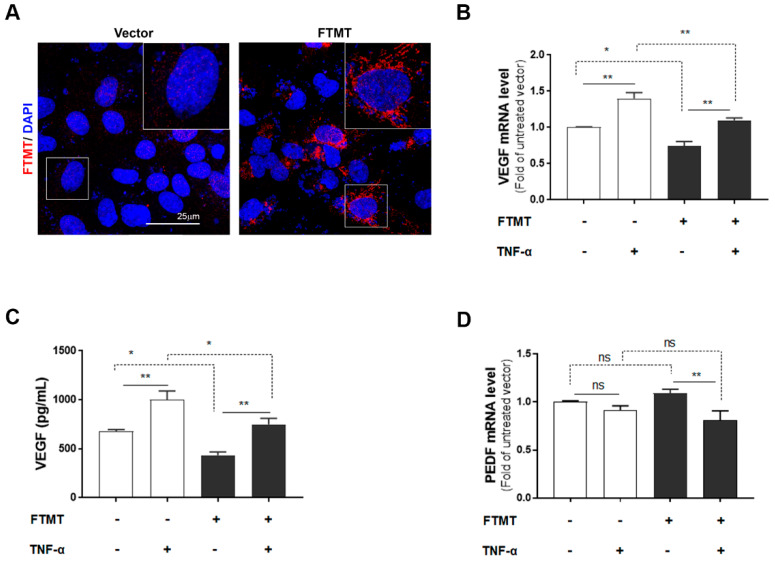
Effects of FTMT overexpression on VEGF secretion in ARPE-19 cells: (**A**) Overexpression of FTMT in ARPE-19 cells. Cells were transfected with pEGFP-N1 vector or FTMT expressing constructs for 48 h. Transfection efficacy was assessed by immunocytochemistry. Scale bar represents 25 μm. FTMT overexpressing ARPE-19 cells were incubated with or without TNF-α (10 ng/mL), and (**B**) the relative mRNA levels of VEGF were analyzed by qPCR and normalized by the ACTB gene. (**C**) VEGF protein expression in the supernatants measured by ELISA. (**D**) The relative mRNA levels of PEDF were analyzed by qPCR and normalized by the ACTB gene using the same samples as in Figure 5B. Data were analyzed by one-way ANOVA with Sidak’s test (* *p* < 0.05; ** *p* < 0.01, *n* = 4). Results are shown as the mean ± SEM.

The above results implied that the activity of FTMT may have an inhibitory effect on VEGF expression and secretion in ARPE-19 cells. To further examine this hypothesis, we transfected ARPE-19 cells with an expression plasmid encoding the human FTMT cDNA. Expression in transfected cells was confirmed by immunocytochemistry (Figure 5A) and Western blot analysis (Supplemental Figure S3A). Efficiency of FTMT transfection was 64% as assessed by the percentage of cells showing immunoreactivity for FTMT antibody. Analysis of vector and FTMT transiently transfected cells showed that increased FTMT resulted in reduced VEGF mRNA and secretion of VEGF protein (Figure 5B,C). This could be observed when comparing columns FTMT−/TNF− with FTMT+/TNF− (Figure 5B,C). There was a decrease in VEGF mRNA expression of 35.4% between these sample groups (Figure 5B). In addition, comparing FTMT− and FTMT+ cells treated with TNF-α, the overexpression of FTMT transfection resulted in significant decrease of VEGF expression. This could be observed when comparing columns FTMT−/TNF+ with FTMT+/TNF+ (Figure 5B,C), where the decrease in VEGF mRNA expression was 27.5%. By comparison, PEDF mRNA expression levels were not significantly affected by FTMT overexpression but were downregulated in ARPE-19 cells overexpressing FTMT and treated with TNF-α (Figure 5D).

The moderate effects of FTMT overexpression using transiently transfected cells on VEGF and PEDF expression requires further investigation but suggests that multiple pathways regulate expression. The finding that TNF-α has effect on cells that are mainly expressing FTMT from the transfected plasmid but also expressing endogenous FTMT suggests that TNF-α might also be increasing plasmid-coded FTMT expression. It has been shown that the plasmid cytomegalovirus immediate early promoter driving FTMT expression can be activated by oxidative stress and NF-κB [39,40].

### 2.6. Feature of Stably Transfected FTMT Overexpressing ARPE-19 Cells

**Figure 6:**
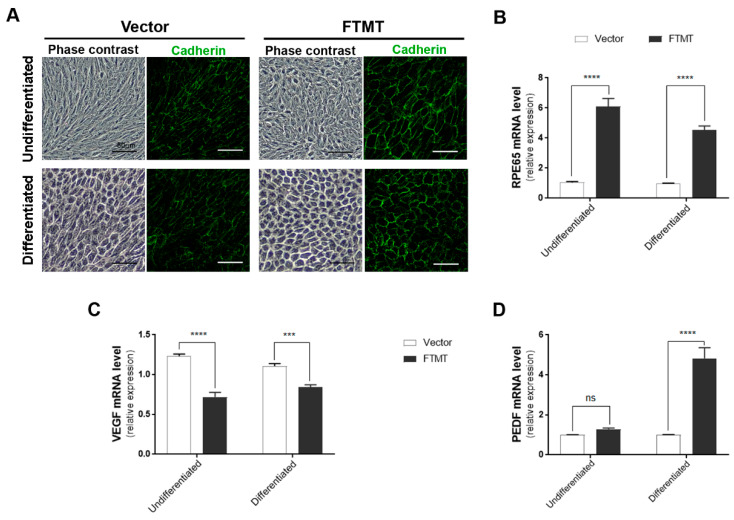
Features of FTMT stably overexpressing undifferentiated and differentiated ARPE-19 cells: (**A**) Morphology (phase contrast) and pan-cadherin immunocytochemistry of control vector transfected and FTMT stably transfected ARPE-19 clones. Scale bars represent 50 μm. (**B**) FTMT overexpressing ARPE-19 clone (undifferentiated and differentiated) expressed significantly higher levels of RPE65 mRNA compared to vector clone. (**C**) FTMT-overexpressing clone (undifferentiated and differentiated) expressed significantly lower levels of VEGF mRNA. (**D**) FTMT-overexpressing clone (differentiated) expressed significantly higher levels of PEDF mRNA. Comparison of relative expression was made between undifferentiated vector and undifferentiated FTMT clone or between differentiated vector and differentiated FTMT clone. Values do not reflect differences between undifferentiated and differentiated cells. Data were analyzed by one-way ANOVA with Sidak’s test, *** *p* < 0.001, and **** *p* < 0.0001, *n* = 4). Results are shown as the mean ± SEM.

The final part of this study involved determining if FTMT overexpression had direct functional effects on angiogenesis. For these studies, we used conditioned media from FTMT overexpressing ARPE-19 cells and vector transfected cells in the widely used endothelial cell in vitro tube formation angiogenesis assay [41]. Firstly, we prepared ARPE-19 cells that stably overexpressed FTMT rather than transiently transfected cells, as these cells provide a stable and consistent level of FTMT protein expression and ensures that all cells are expressing FTMT. Stable FTMT overexpressing cells also allowed comparison of effects of FTMT and differentiation on angiogenesis-related and cellular properties. Figure 6A shows the different morphologies of vector transfected and FTMT transfected ARPE-19 cells, both undifferentiated and differentiated cells. It can be seen that differentiation in nicotinamide-containing media produced similar cobblestone morphology for both types but that the FTMT overexpressing cells had a more mature morphology with increased expression of cadherin junction protein.

Supplemental Figure S3B shows the western blot levels of FTMT protein in FTMT transfected cells (red numbers) compared to control vector cells (black numbers). The highest FTMT expressing clone (#2) and one control vector clone were expanded for use in these studies. Only a single high FTMT expressing clone could be isolated, but levels of FTMT expression in this clone appeared stable over a limited number of passages. Similar to what was observed in Figure 5A, high levels of FTMT expression did not affect the growth and viability of cells. An interesting feature between the FTMT stable clone and vector stable clone was observed for expression of RPE65 mRNA, a differentiation marker for RPE cells. Figure 6B shows the relative differences between undifferentiated cells, where the FTMT overexpressing clone had 4.6-fold greater expression of RPE65 mRNA, while differentiated FTMT overexpressing cells had 4.3-fold greater expression. The increased expressions due to differentiation for vector transfected cells were 15.6-fold and 14.7-fold for FTMT transfected cells. FTMT-overexpressing cells expressed significantly less VEGF mRNA (38.1% in undifferentiated cells to compare to transient transfected cells (Figure 6C)) and, if differentiated, had significantly increased PEDF mRNA (Figure 6D).

### 2.7. Conditioned Media from FTMT-Overexpressing Cells Inhibit Endothelial Cell Growth and Tube Formation

Conditioned media was prepared from stable FTMT overexpressing and vector transfected clones from both undifferentiated and differentiated cultures. Conditioned media was collected after 2 days.

We employed the hCMEC/D3 cerebrovascular endothelial cell line as the indicator cell type in the in vitro tube formation assay and cell proliferation assay [42,43]. This cell line originated from endothelial cells from cerebral microvessels and was transformed by SV40 T antigen and human telomerase (hTERT) and is the human endothelial cell line we have found to give consistent performance in the in vitro angiogenesis tube formation assay. Experiments were carried out with media from FTMT overexpressing and vector transfected cells and were used undiluted (100) or diluted 50:50 with original media. Preliminary analysis using a cell viability assay showed that hCMEC/3D exposed to conditioned media samples for 24 h did not have evidence of toxicity (Supplemental Figure S4).

**Figure 7:**
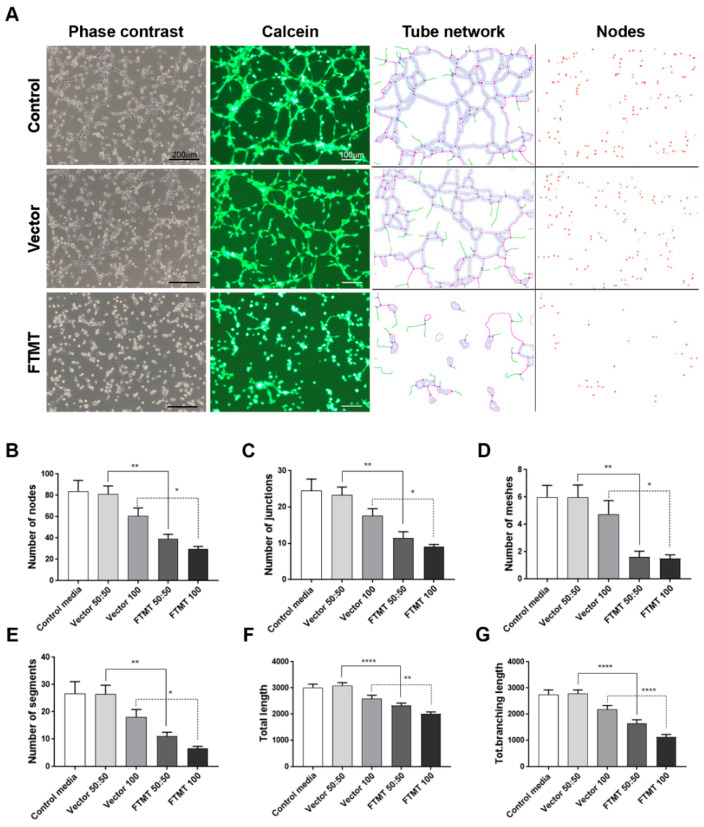
Conditioned media from undifferentiated FTMT-overexpressing ARPE-19 cells inhibit in vitro angiogenesis. (**A**) Representative phase contrast micrographs and calcein-AM fluorescent images of hCMEM/3D endothelial cell tube formation after 6 h in indicated condition media samples: Examples of ImageJ processed images for quantification of tube networks and nodes. Scale bars on phase contrast images represent 200 μm and, on Calcein images, represent 100 μm. (**B–G**) Bar charts showing effects at 6 h on hCMEM/3D tube formation parameters of control media, undiluted (vector 100) and diluted (vector 50:50) vector transfected cell conditioned media, and undiluted (FTMT 100) and diluted (FTMT 50:50) FTMT transfected cell conditioned media. (**B**) Number of tube nodes, (**C**) number of tube junctions, (**D**) number of tube meshes, (**E**) number of tube segments, (**F**) total tube length, and (**G**) total tube branching length. Results represent mean + S.E.M. of 2 replicate experiments with 4 measurements per treatment (*n* = 8). Data were analyzed by one-way ANOVA with Sidak’s test (* *p* < 0.05; ** *p* < 0.01, and **** *p* < 0.0001, *n* = 8).

**Figure 8:**
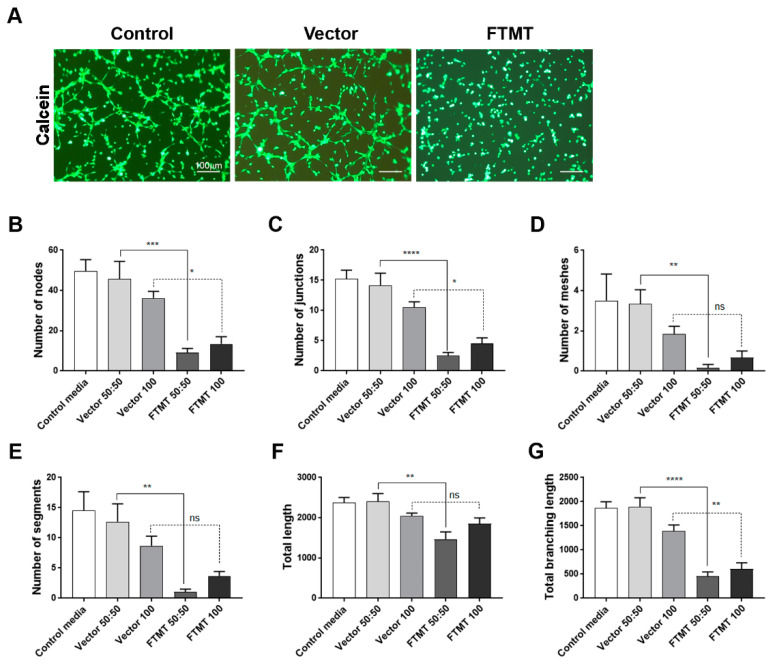
Conditioned media from differentiated FTMT-overexpressing ARPE-19 cells inhibit in vitro angiogenesis: (**A**) Representative calcein-AM fluorescent images of hCMEM/3D endothelial cell tube formation after 6 h in indicated condition media samples. Scale bars represent 100 μm. (**B**–**G**) Bar charts showing effects at 6 h on hCMEM/3D tube formation parameters of control media, undiluted (vector 100) and diluted (vector 50:50) vector transfected cell conditioned media, and undiluted (FTMT 100) and diluted (FTMT 50:50) FTMT transfected cell conditioned media. Results represent mean + S.E.M. of 2 replicate experiments with 4 measurements per treatment (*n* = 8). Data were analyzed by one-way ANOVA with Sidak’s test (* *p* < 0.05; ** *p* < 0.01, *** *p* < 0.001, and **** *p* < 0.0001, *n* = 8).

Results of angiogenesis tube assays are shown in Figure 7 (cell media from undifferentiated cells) and Figure 8 (cell media from differentiated cells). Images of calcein-AM green fluorescent tubes recorded at 6 h were analyzed using the Angiogenesis Plug-In for ImageJ analysis software. Examples of the computer-adjusted images to detect tube network and nodes are shown in Figure 7A (tube network and nodes). The different types of angiogenesis/tube formation measurements are presented (Figure 7B–G). Similar measurements were made using endothelial cells incubated with media from differentiated cells (Figure 8). Overall findings show that conditioned media from FTMT-overexpressing cells significantly inhibited in vitro tube formation whether derived from undifferentiated (Figure 7) or differentiated (Figure 8) cells. Statistical analyses were carried out by comparing Vector 50:50 to FTMT 50:50 and Vector 100 to FTMT100.

We also examined the effect of conditioned media from FTMT-transfected and vector transfected cells on hCMEC/D3 proliferation over 48 h. FTMT-transfected cell-conditioned media from undifferentiated cells significantly inhibited proliferation of cells compared to control and vector-transfected cell-conditioned media (Supplemental Figure S5).

## 3. Discussion

This study identified features related to angiogenesis expressed by the RPE cell line, ARPE-19, that were affected by modulating the expression of FTMT. We showed significant changes in cellular properties of ARPE-19 cells upon differentiation to acquire features of tissue RPE cells. These differences should be considered when a goal is to employ in vitro models relevant for human diseases such as AMD. In addition, this study focused on FTMT effects on separate features of angiogenesis, namely VEGF expression, PEDF expression, and endothelial tube formation as a direct assay for angiogenesis.

Controlling aberrant angiogenesis, the formation of new vessels under pathological conditions, is an important therapeutic target for cancer, diabetes, stroke, and retinal diseases, among others [44,45,46,47,48]. Preventing neovascularization in many types of cancers is important as it reduces the aerobic and nutritional supplies that promote the growth of cancerous cells. In diabetes and stroke, the other forms of aberrant angiogenesis are encountered where the formation of new vessels needed to support damaged tissues becomes inhibited. In retinal diseases associated with diabetes and AMD, both forms of aberrant angiogenesis can be encountered. Angiogenesis is a complex coordinated procedure that requires a number of different regulatory growth factors along with the involvement of different cell types; however, VEGF is the most widely studied angiogenic factor. VEGF is frequently targeted to prevent neovascularization, with neutralizing antibodies or VEGF receptor antagonists being tested at the clinical level [44]. Another therapeutic target is the anti-angiogenic cytokine PEDF where the increased expression of PEDF or use of PEDF-derived small peptides has significant protective effects in retinal neovascularization diseases [49,50,51]. The importance of understanding neovascularization mechanisms in AMD was one of the rationales for this study. An inhibitory role for FTMT would suggest the involvement of oxidative stress mechanisms in the regulation of VEGF and possibly PEDF.

Effects of FTMT overexpression on phenotypes of differentiated and undifferentiated ARPE-19 cells were identified. This cell line has been widely used in vision research projects, particularly in investigations of mechanisms of age-related macular degeneration (AMD). The use of continuously cultured cell lines for research offers convenience compared to preparation of tissue-derived primary cells, but mature differentiation markers can be lost from cell lines with continuous culture. This has been observed in ARPE-19 cells, but strategies for preserving or inducing expression of genes associated with differentiated RPE cells have been identified. Culturing of ARPE-19 cells for 3–4 weeks in low-serum media resulted in expression of differentiation-associated genes [36], but a more rapid method that induced a differentiated phenotype by culture of cells in nicotinamide-containing media for 7–10 days was recently discovered [37]. This latter method was applied in this study and showed that differentiated ARPE-19 cells expressed over 300-fold greater levels of mRNA for the RPE signature gene RPE65 (Figure 1). We also observed that ARPE-19 cells stably overexpressing FTMT but maintained under undifferentiated conditions expressed approximately 5-fold greater RPE65 mRNA than vector transfected cells (Figure 6). This could be related to the antioxidant properties of FTMT, but further studies are needed. Differentiated ARPE-19 cells treated with proinflammatory cytokines, which can induce cellular oxidative stress, or hydrogen peroxide expressed significantly lower levels of RPE65 mRNA [36,52,53]. Differentiated ARPE-19 cells constitutively expressed less VEGF mRNA. Mutations in RPE65 have been associated with a number of inherited retinal dystrophies (IRD) [54,55]. RPE65 gene therapy for these predominantly juvenile diseases have shown promising results, but the mechanism of how loss of function of RPE65, the enzyme retinoid isomerohydralase, results in retinal degeneration is not known [56]. Transplantation of RPE65 transduced bone marrow-derived monocytes prevented progression of chronic retinal degeneration in an animal model [57]. The link with FTMT requires further investigation, but RPE65 is a non-heme iron-dependent monotopic protein that converts 1-cis-retinal to all-trans-retinal in the phototransduction pathway.

We also demonstrated that proinflammatory cytokines TNF-α, IL-1β, and IFN-γ induced FTMT and VEGF transcription in ARPE-19 cells and VEGF secretion. As increased VEGF promotes angiogenesis in a number of retinal diseases, such as AMD and diabetic retinopathy, it has made VEGF a highly significant therapeutic target [6,9,46]. Induction of VEGF in retinal cells can result from age-associated oxidative stress and inflammation [15,58]. Proinflammatory cytokines, such as TNF-α are involved in the pathogenesis of many eye conditions, including ocular inflammation, edema, choroidal neovascularization, and neurodegenerative disorders [14,59,60]. A number of studies have suggested a possible role of TNF-α in aging and AMD-related RPE changes being caused by age-associated increases of TNF-α [61,62]. We investigated whether TNF-α-upregulated expression of FTMT and VEGF secretion could be inhibited by NF-κB activation. Our data showed that TNF-α-upregulated FTMT expression was inhibited by an NF-κB inhibitor, BAY 11-7082, similar to our previous study using IMR-32 neuroblastoma cells [28].

FTMT gene silencing increased VEGF secretion compared to the control group. In contrast, the overexpression of FTMT served to reduce VEGF mRNA and protein expression in both TNF-α treated and untreated cells. Our results suggested that FTMT has an inhibitory effect on VEGF secretion in ARPE-19 cells. However, as the overexpression of FTMT did not abolish TNF-α induced increase in VEGF mRNA and protein secretion, this would suggest that the link between FTMT and VEGF could be indirect through multiple signaling mechanisms.

Finally, we were also able to demonstrate using an in vitro angiogenesis tube formation assay that an altered pattern angiogenic factors from FTMT-overexpressing cells significantly inhibited most of the in vitro features of angiogenesis measured. To be able to study the effect of FTMT, we produced stably transfected FTMT overexpressing cells rather than rely on less stable and less-efficient transient overexpression. As mentioned above, the increased expression of FTMT significantly increased the expression of RPE65, but we also observed decreased expression of VEGF, in agreement with other results, and increased expression of PEDF mRNA. The in vitro angiogenesis tube formation assay encompasses multiple features required for endothelial cells to form into vessel-like structures, including adhesion, migration, and tubule formation in a coordinated manner depending on the balance of pro-angiogenic and antiangiogenic factors [63]. This assay has been used to screen for inhibitors and to identify signaling mechanisms involved (for examples, see References [64,65,66]). We have focused mainly on VEGF and to a less extent on PEDF, as effectors of angiogenesis and angiogenesis inhibition, but a number of factors are involved to produce the end result of vessel formation. Further studies using proteomics approaches to screen the media from FTMT-overexpressing and control cells to identify the differential angiogenic factor secretome are suggested. This will give a complete picture of the different factors involved. However, based on the current state of knowledge of the multiple features of FTMT, one can hypothesize that its potent antioxidant properties could modulate gene expression of angiogenic and inflammatory factors in RPE cells even under normal conditions. Future studies can use the cells and assays developed for this study to determine the effect of FTMT under pathological/oxidative stress/inflammatory conditions.

In conclusion, our results demonstrated that FTMT has an inhibitory effect on VEGF expression and secretion in ARPE-19 cells; alters the phenotypes of overexpressing cells; and alters the secreted angiogenic factors from overexpressing cells, resulting in inhibition of angiogenesis.

## 4. Materials and Methods

### 4.1. Cell Culture

The human retinal pigment epithelium cell line, ARPE-19, was obtained from the American Type Culture Collection (Manassas, VA, USA) [34]. Cells were cultured in Dulbecco’s modified Eagle’s medium-F-12 nutrient mixture (DMEM/F-12; Nacalai-Tesque, Kyoto, Japan) with 10% fetal bovine serum (FBS; Corning, Manassas, VA, USA), 100 U/mL penicillin, and 100 U/mL streptomycin. Cells were cultured in 100 mm dishes (1.2 × 10^6^ cells) and incubated in a humidified incubator at 37 °C in 5% CO₂. The culture media was exchanged every 3–4 days, and the cells were passaged every 6–8 days. Trypsin EDTA (Nacalai-Tesque) was used to detach the cells after they reached confluence.

ARPE-19 cells were differentiated according to a previously published method with modifications [37]. Briefly, ARPE-19 cells were seeded to the 6-well plate (5 × 10^5^ cells/well), and cells were grown in the following media for 10 days: MEM alpha medium with supplements including 10 mM nicotinamide, 1% FBS, 1% gentamicin, 0.1 mM NEAA (Invitrogen), 1% N1 supplement (Sigma-Aldrich), taurine (0.25 mg/mL), hydrocortisone (20 ng/mL), and triiodo-thyronine (0.013 ng/mL)(Nacalai-Tesque). The cells were incubated at 37 °C in 5% CO₂, and the media was replaced with fresh media every three days.

The human cerebrovascular endothelial cell line hCMEC/D3 [42] was obtained from Millipore/Sigma (Burlington, MA, USA) and cultured in Endothelial Basal Cell Media (EBM-2) with microvascular endothelial cell growth supplements (Lonza Biosciences, Walkerville, MD, USA). Cells were cultured on collagen-type 1-C coated flasks (0.3 mg/mL) (Cell-Matrix-Kurabo Industries, Osaka, Japan). Cells were subcultured with 0.125% trypsin/EDTA before reaching confluence. For use in the in vitro tube formation assay, hCMEM/D3 cells were passaged no more than 3 times.

### 4.2. Cell Treatments

ARPE-19 cells were cultured in 2 mL DMEM/F-12 containing 10% FBS in 6-well plates at a density of 4 × 10^5^ cells/well. After cells had attached (18–24 h), the media was replaced with serum-free DMEM/F-12 media containing 10 ng/mL of human IL-1β, 10 ng/mL of TNF-α, or 100 U/mL of IFN-γ (PeproTech, Rocky Hill, NJ, USA). Proinflammatory cytokines were tested at various concentrations (0.5–50 ng/mL) in ARPE-19 cells, and optimal doses were chosen (data not shown).

In order to examine the involvement of NF-κB signaling pathway, cells were pre-incubated with NF-κB inhibitor BAY 11–7082 (Calbiochem, San Diego, CA, USA) at a concentration of 5 µM in 2 mL serum-free media for 30 min before treatment with 10 ng/mL of TNF-α. An equal volume of DMSO was used as the untreated control. After incubation for 24 h, the total RNA was isolated for quantitative real-time reverse transcription polymerase chain reaction (qPCR). The media supernatants were stored at −80 °C until further use.

### 4.3. Plasmid Transfection

Cells were transfected with the FTMT expression construct and corresponding empty vector for which construction and characterization have been previously described [67]. Briefly, cells were seeded at 2 × 10^5^ cells/well in 6-well plates. After 24 h incubation, the media was replaced with OPTI-MEM I reduced serum medium (Invitrogen, Life Technologies) and 1 µg of plasmid DNA was transfected into cells using Lipofectamine 3000 (Invitrogen, Life Technologies) according to the manufacturer’s instructions. After 12 h, serum was added to the media to a final concentration of 5% and cells were incubated for another 12 h. The media was then removed, and 2 mL of fresh growth media was added. After 48 h transfection, cells were stimulated with TNF-α with or without the BAY 11-7082 inhibitor, as described above, and specific experiments were performed with treated cells.

To isolate FTMT and vector stable transfected clones, ARPE-19 cells transfected as described above were plated to petri dishes in DMEM/F-12 + 10% FBS with 500 μg/mL G418 as selection media (Nacalai-Tesque, Kyoto, Japan). The cells were incubated under selective conditions, and media was refreshed three times in a week. Twelve separate G418-resistant colonies that grew out for each vector were cloned out and expanded independently. Each clone was analyzed by western blot for expression of FTMT protein. The highest expressing FTMT clone was selected for further experimentation.

### 4.4. FTMT Gene Silencing by Small Interfering RNA (siRNA)

Cells were transfected with Silencer-Select pre-designed FTMT siRNA (ID: s41238) obtained from Ambion (ThermoFisher) or the Stealth RNAi negative control using Lipofectamine RNAiMAX reagent (Invitrogen, Life Technologies) according to the manufacturer’s instructions. Briefly, cells were seeded at a concentration of 2 × 10^5^ cells/well in 6-well plates. After 24 h, the media was replaced with serum-free DMEM/F-12, and cells were transfected with 20 pmol of siRNA or negative control using the transfection reagent (5 μL per well) and were incubated for 48 h. After transfection, some of the cultures were treated with TNF-α (10 ng/mL), as described above, for specific experiments. The efficiency of gene silencing was evaluated by qPCR.

### 4.5. RNA Extraction and Real-time Reverse Transcription-Polymerase Chain Reaction (qPCR)

Total RNA was extracted from the cell lysates by using the RNeasy Plus Mini kit (Qiagen, Germany) as described by the manufacturer. The RNA yield was determined using a Nanodrop spectrophotometer (Thermo Scientific). An equal amount of RNA (0.5 to 0.7 µg total cellular RNA) was reverse transcribed using the PrimeScript RT kit with genomic DNA eraser (Takara Bio, Japan). qPCR analysis of FTMT and VEGF expressions was carried out using the THUNDERBIRD SYBR Green qPCR Mix (Toyobo, Osaka, Japan). The ACTB gene transcript was used as a housekeeping control to normalize the transcripts in each sample.

The sequences of the primers used in this study were as follows: FTMT 1, forward -5′- CCATTTGTGCGATTTCCTG -3′; reverse -5′- GCTCGTGGCTTAGTTCTGCT -3′; VEGF, forward -5′- TTGCCTTGCTGCTCTACCTC -3′; reverse -5′- AAATGCTTTCTCCGCTCTGA -3′; ACTB, forward -5′- CCTATGTGGGCGACGAG -3′; reverse -5′- ATGGCTGGGGTGTTGAAG -3′; RPE65, forward -5′- TGCGTATGGACTTGGCTTG -3′; reverse -5′- GCTCACCACCACACTCAGAA -3′; PEDF, forward -5′- GTGGCACCTCTGGAAAAGTC -3′; reverse -5′- ACCGAGAAGGAGAATGCTGA -3′. The primers were used at a concentration of 12.5 pmol/reaction, and each sample was analyzed in duplicate using triplicate biological replicates with a Roche Light Cycler 480 qPCR machine. QPCR results are expressed as fold changes in expression relative to untreated control cells.

### 4.6. Nuclear and Cytoplasmic Extracts and Western Blot Analysis

To examine the efficiency of BAY 11-7082 treatment on inhibition of NF-κB signaling, cells were incubated either in the absence or presence of BAY 11-7082 (5 μM) and TNF-α (10 ng/mL) for 24 h. Nuclear and cytoplasmic protein fractions were extracted using the NE-PER Nuclear and Cytoplasmic Extraction Reagent Kit (Thermo Scientific) according to the manufacturer’s protocol. Total extracts were then prepared and subjected to SDS-PAGE and western blot analysis.

Cell lysates were sonicated, and protein concentrations were determined using Micro-BCA Protein Assay Kit (Thermo Scientific). Samples were diluted in SDS-loading buffer and heated at 95 °C for 10 min. Equal amounts of protein were then loaded onto 15% SDS gradient gels (Wako, Japan). The gels were electroblotted onto PVDF membranes (Millipore, MA, U.S.A.) and membranes blocked with 4% skim milk for 1 h. The membranes were then incubated overnight at 4 °C with the following primary antibodies in TBST containing 0.003% sodium azide: mouse-anti-FTMT (1:3000; [31]), rabbit-anti-NF-κB p65 (1:1000; ab7970, Abcam, Cambridge, MA, USA), rabbit-anti-histone-H3 (1:10000; ab21054, Abcam, Cambridge, USA), and mouse-anti-β-actin (1:5000; sc-47778, Santa Cruz Biotechnology, CA, USA). After washing with TBST, the membranes were incubated with HRP- labeled anti-mouse or anti-rabbit secondary antibody (1:10000; Jackson ImmunoResearch Laboratories, Inc., West Grove, PA, USA) in 2% skim milk in TBST at room temperature for 1 h. Signals were visualized with the ChemLumi One HRP chemiluminescence substrate (Nacalai-Tesque) in conjunction with LAS 4000 image analyzer (GE Biosciences, Chicago, IL, USA).

### 4.7. Immunocytochemistry

ARPE-19 cells were plated onto chamber slides, and then cells were fixed with 4% paraformaldehyde for 20 min and blocked with 5% skim milk for 1 h at room temperature. Cells were then incubated with the rabbit-anti-FTMT primary antibody (1:500) or mouse anti-pan cadherin antibody (1:200) (ab6528-Abcam, Cambridge, U.K.) overnight at 4 °C. After washing three times with TBST, cells were incubated with Alexa Fluor 568-conjugated anti-rabbit immunoglobulin G (1:1000; ThermoFisher) to detect FTMT or with Alexa Fluor 488-conjugated anti-mouse immunoglobulin G (1:1000; ThermoFisher) for 1 h at room temperature; then, cells were counterstained with DAPI to allow for the identification of nuclei and cover-slipped using a fluorescent-mounting agent. Stained cells were examined using a confocal microscope (Leica SP8 Lightning, Leica, Wetzlar, Germany).

### 4.8. Enzyme-Linked Immunosorbent Assay (ELISA)

The cell culture supernatants with or without proinflammatory cytokines and NF-κB inhibitor were collected for determining concentration by using the human ELISA development kit according to the manufacturer’s instruction (Quantikine Human VEGF Immunoassay ELISA Kit; R&D Systems, Minneapolis, MN, USA).

### 4.9. Preparation of Transfected Cell-Conditioned Media

For preparation of conditioned media from undifferentiated vector and FTMT plasmid transfected ARPE-19, cells were seeded at 4 × 10^5^ cells/well in 6-well plate with DMEM/F-12 containing 10% FBS and cultured overnight. The medium was exchanged with serum-free DMEM/F-12 and incubated for 48 h. For preparation of conditioned media from differentiated cells, Vector and FTMT plasmid transfected ARPE-19, cells were seeded at 4 × 10^5^ cells/well in 6-well plate with DMEM/F-12 containing 10% FBS and cultured overnight. The media was exchanged for differentiation media, and cells were prepared according to protocol (Section 4.1). The medium was exchanged with serum-free MEM-alpha and incubated for 48 h. The conditioned medium was collected and stored in a −80 °C freezer. The control medium was serum-free DMEM/F-12 (undifferentiated) or serum-free MEM alpha (differentiated) that had not been exposed to cells.

### 4.10. Cell Viability Assay

Cell viability was determined using the WST--1 reagent (Cell Count Reagent; Nacalai-Tesque) according to the manufacturer’s protocol. Target cells (ARPE-19 or hCMEC/D3) were plated at 1.5 × 10^4^ cells/well in 96-well microtiter plates. ARPE-19 cells were treated for 24 h with NF-κB inhibitor or cytokines, or hCMEMC/D3 cells were treated with diluted conditioned media for 24 h. Cell viability reagent (10 μL) was added to each well for 4 h, and then, the resulting color change was measured using a Tecan-2000 plate reader at 450 nm wavelength. Relative changes in absorbance were determined after subtracting the absorbance measured in cell-free wells. Cell viability was then calculated as percent changes relative to DMSO-treated or control media-treated control cells.

### 4.11. In Vitro Tube Formation Assay

An in vitro angiogenesis assay, the cell tube formation assay, was carried out to detect whether FTMT expression affected angiogenesis. The procedure used was a modification of published protocols [41,68,69,70] adapted for use with microtiter plates. Endothelial cells (hCMEC/D3) were grown as described in Section 4.1 but, 24 h before use in angiogenesis assay, were cultured in DMEM media containing 0.2% FBS. Cells were labeled with calcein-AM (Sigma-Aldrich) at a final concentration of 2 μg/mL for 30 min. Cells were removed from culture vessel with 0.125% Trypsin/1 mM EDTA, centrifuged at 200 g/3 min, and resuspended in DMEM/0.2% FBS. For this assay, 24 wells of a 96-well microplate were coated with growth factor-reduced matrigel (60 μL/well) (Corning, NY, USA) and allowed to polymerize for 30 min at 37 °C. The wells used were equivalent distances from the edge of the plate to ensure equal gas exchange. Calcein-AM-labeled endothelial cells were seeded at 3 × 10^4^ cells/well into the matrigel-coated wells of the microtiter plate in 100 μL conditioned media of FTMT-overexpressing or vector-transfected ARPE-19 cells. The optimal cell number for this assay was determined in preliminary experiments (data not shown). Conditioned media from undifferentiated cells was used undiluted (100) or diluted 1:1 (50:50) in serum-free DMEM/F12. Conditioned media from differentiated cells was used undiluted (100) or diluted 1:1 (50:50) in serum free MEM-alpha.

Images were taken with fluorescent and light microscopes (Olympus, Tokyo, Japan) 6 h after seeding. Tube formation from four randomly selected fields of images from each treatment were analyzed by NIH Image J with the Angiogenesis Analyzer plugin, based on different parameters such as number of nodes, junctions, meshes, segments, branching length, and total length using described analysis protocols [41]. Results represent the mean data combined from duplicate independent experiments (*n* = 8).

### 4.12. Statistical Analysis

All results are expressed as the mean ± SEM. Statistical analyses were performed using GraphPad Prism 7 software (GraphPad, La Jolla, CA, USA). Experimental data were analyzed by one-way ANOVA followed by Dunnet’s or Sidak’s tests of multiple comparisons to examine the significance among groups. Values of *p* < 0.05 were considered to be significantly different.

## Supplementary Materials

Supplementary Materials can be found at https://www.mdpi.com/1422-0067/21/10/3635/s1.
