# Supplementary material for: Effects of FTMT Expression by Retinal Pigment Epithelial Cells on Features of Angiogenesis
Source: Int J Mol Sci. 2020 May 21;21(10):3635. doi: 10.3390/ijms21103635 (PMC7279371; doi:10.3390/ijms21103635)
Supplement: Supplementary file 1 [file ijms-21-03635-s001.pdf]

Supplementary Materials:

Effects of FTMT Expression by Retinal Pigment Epithelial Cells on Features of Angiogenesis

Undral Buyandelger, Douglas G. Walker, Daijiro Yanagisawa, Toshifumi Morimura and Ikuo Tooyama *

Molecular Neuroscience Research Center, Shiga University of Medical Science, Seta Tsukinowa-cho, Otsu, Shiga 520-2192, Japan; undral@belle.shiga-med.ac.jp (U.B.); walker@belle.shiga-med.ac.jp (D.G.W.); yanisagawa@belle.shiga-med.ac.jp (D.Y.); morimura@belle.shiga-med.ac.jp (T.M.)

* Correspondence: kinchan@belle.shiga-med.ac.jp; Tel.: +81-77-548-2330; Fax: +81-77-548-2331

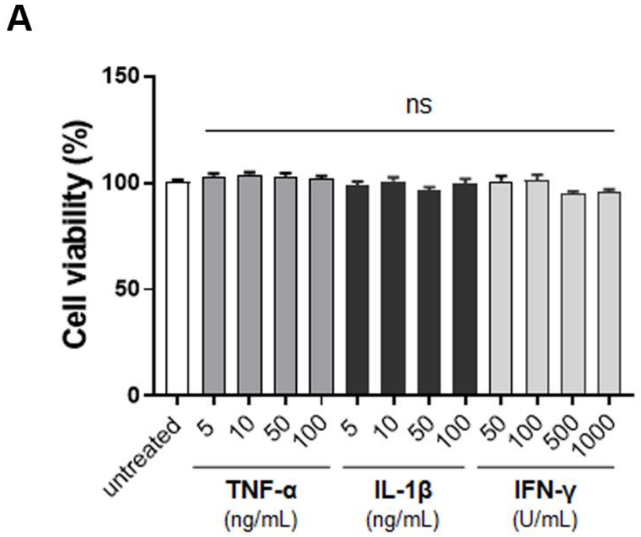

Supplemental Figure S1. Cell viability assay to show effect of pro-inflammatory cytokines on ARPE-19 cells.

ARPE-19 cells plated at 1.5 × 10^4 cells/well in microtiter plate and were treated with indicated concentrations of pro-inflammatory cytokines for 24 hours followed by addition of Cell-Count Reagent (Nacalai-Tesque, Kyoto, Japan) (WST-8) for 4 hours. Absorbance recorded at 450 nM. Results represent mean ± S.E.M. of 8 replicate wells/treatment. Data were analyzed by One-way ANOVA with Sidak's test (ns-non significant difference).

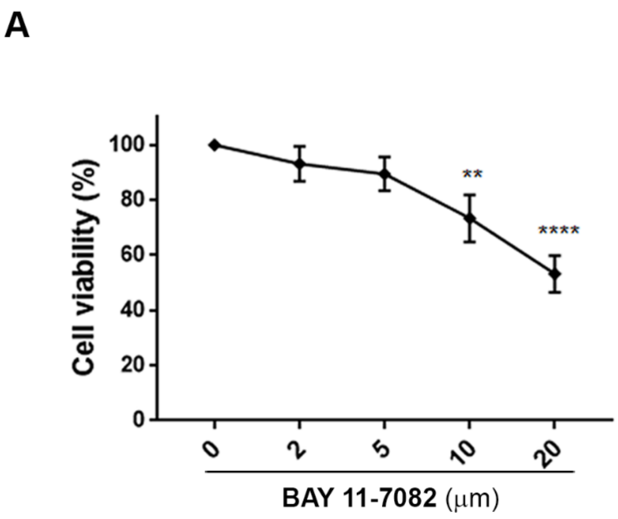

Supplemental Figure S2. Cell viability assay to show effect of different concentrations of NF-κB inhibitor BAY 11-7082 on ARPE-19 cells.

ARPE-19 cells plated at 1.5 x 10^4 well in microtiter plate were treated with indicated concentrations of BAY 11-7082 for 24 hours followed by addition of Cell-Count Reagent (Nacalai-Tesque, Kyoto, Japan) (WST-8) for 4 hours. Absorbance recorded at 450 nM. Results represent mean $\pm$ S.E.M. of 6 replicate wells/treatment. Data were analyzed by One-way ANOVA with Sidak's test (** $p < 0.01$, **** $p < 0.0001$, $n = 6$).

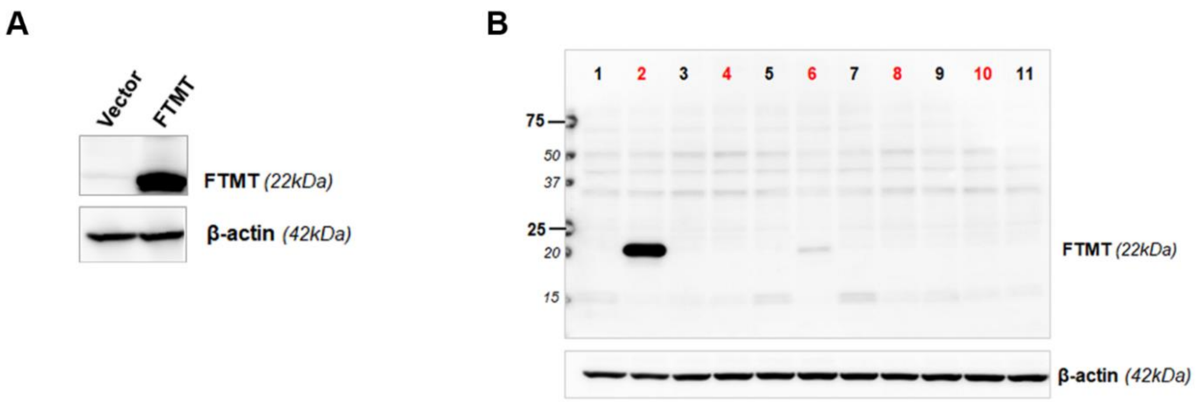

Supplemental Figure S3. Western blot screening of FTMT transfected ARPE-19 cells.

(**A**). Western blot showing expression of FTMT in transiently transfected cells (control vector and FTMT plasmid vector). Protein extracts from vector transfected and FTMT transfected cells were separated by SDS-PAGE and analyzed for expression of FTMT protein by western blot.

(**B**) Western blot screening of stably transfected ARPE-19 cells isolated after G418 selection. Protein extracts from vector transfected (black numbers) and FTMT transfected clones (red numbers) were separated by SDS-PAGE and analyzed for expression of FTMT protein by western blot. The highest expressing FTMT clone (2) was expanded.

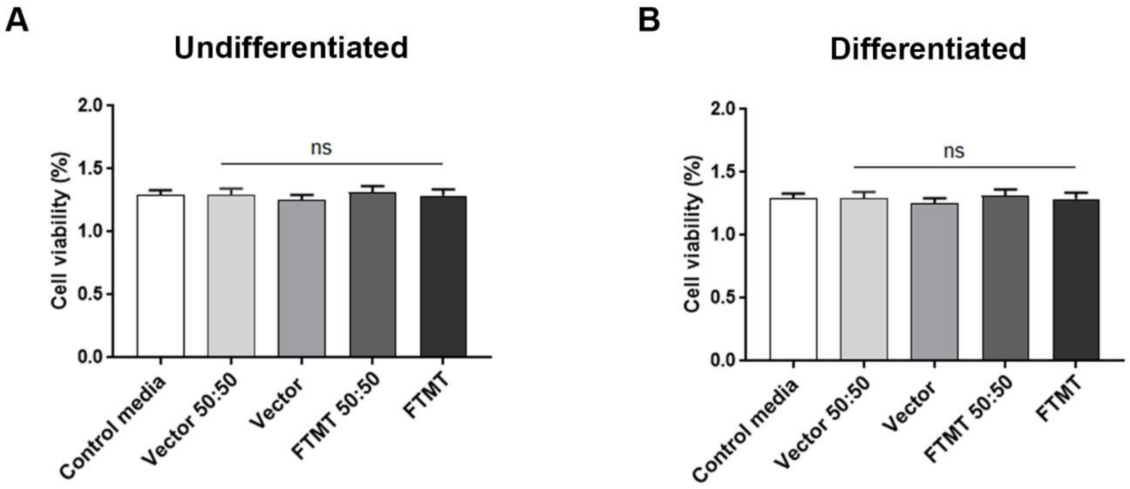

Supplemental Figure S4. Cell viability assay to test for toxicity of FTMT-transfected and vector-transfected conditioned media on hCMEC/D3.

hCMEC/D3 plated at 1.5 × 10^4 well in microtiter plate were treated with indicated dilutions of conditioned media from vector and FTMT stably transfected ARPE-19 cells for 24 hours followed by addition of Cell-Count Reagent (WST-8) for 4 hours. Absorbance recorded at 450 nM. Results represent mean $\pm$ S.E.M. of 6 replicate wells/treatment. Data were analyzed by One-way ANOVA (ns-non-significant differences)

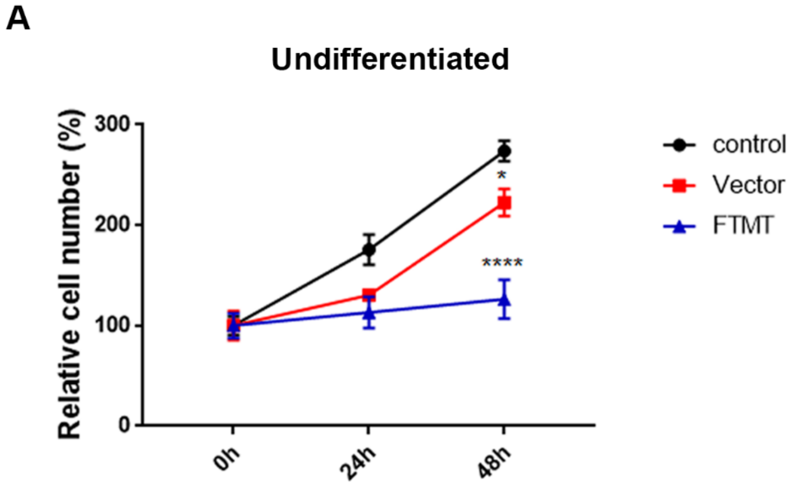

Supplemental Figure S5. Effect of conditioned media from FTMT-transfected or vector-transfected ARPE-19 cells on cell proliferation of hCMEC/D3 endothelial cells.

Effect of ARPE-19 conditioned media from undifferentiated ARPE-19 cells stably transfected with vector or FTMT plasmids. Conditioned media was diluted 1:1 with EC growth media. hCMEC/D3 cells were plated at 2.5 × 10^4 cells/well on a 24-well tissue culture plate. After 6 hours to permit attachment, growth media was replaced with diluted conditioned media from Vector or FTMT-transfected ARPE-19 cells. Phase contrast images were obtained at 0 h, 24 h and 48 h and analyzed for percentage area of field occupied by cells using ImageJ analysis software. Data were analyzed by One-way ANOVA with Sidak's test (* $p < 0.05$, **** $p < 0.0001$, $n = 4$).
